# Left Ventricular Outflow Occlusion: An Alternative Approach to Late Selective Fetal Termination in Monochorionic Twin Gestations

**DOI:** 10.7759/cureus.41974

**Published:** 2023-07-16

**Authors:** Richard N Brown, Anne-Maude Morency

**Affiliations:** 1 Obstetrics and Gynecology, McGill University, Montreal, CAN

**Keywords:** high risk obstetrics, endovascular occlusion, selective termination, discordant anomaly, monochorionic twins, twin pregnancy

## Abstract

We report the case of a monochorionic twin gestation discordant for a mutation in the chromodomain-helicase-DNA-binding protein 7 (CHD7) gene and cerebral abnormalities consequent to an early devastating cerebrovascular event. The parents elected for selective termination given the poor prognosis for this fetus, but given socio-economic considerations wished to defer this procedure as late in gestation as possible, despite awareness of the risks and limitations of existing techniques at the end of pregnancy.

A novel technique was used to achieve selective feticide in the late-preterm period. An endovascular balloon catheter was used to occlude the left ventricular outflow and coronary circulations resulting in fetal asystole while also arresting fetoplacental flow in this fetus, immediately prior to the delivery of the healthy fetus.

## Introduction

Monochorionic twins discordant for fetal anomalies can present clinical dilemmas with respect to management approaches with the diagnosis, gestation at diagnosis, and prognosis of abnormalities found being principal considerations. Monochorionic gestations are characterized by the presence of intertwin vascular communications preventing the use of cardioplegic approaches as used in di-chorionic gestations undergoing selective reduction. Death of a single fetus in a monochorionic gestation may pose risks of death or neurological injury to the surviving twin due to these intertwin vascular communications. Therefore, selective termination in monochorionicity relies upon a vaso-ablative approach, occlusion of the cord at its abdominal insertion or along its length, that minimizes the possibility of flow across these vascular communications by interrupting blood flow to and from the body of the targeted fetus, thereby minimizing the risk of harm to the co-twin following fetal death. Radiofrequency (RF) and bipolar cord occlusion are presently the most widely used approaches [1-3]. Selective reduction carries the risk of complications including miscarriage or preterm delivery, depending upon the gestation at which this is performed and although latency to delivery is overall good there are still risks of significant prematurity and pregnancy loss. Most series describe cases being undertaken at 30 weeks of gestation or less with very few reported cases beyond this [1,4]. 

## Case presentation

**Figure 1:**
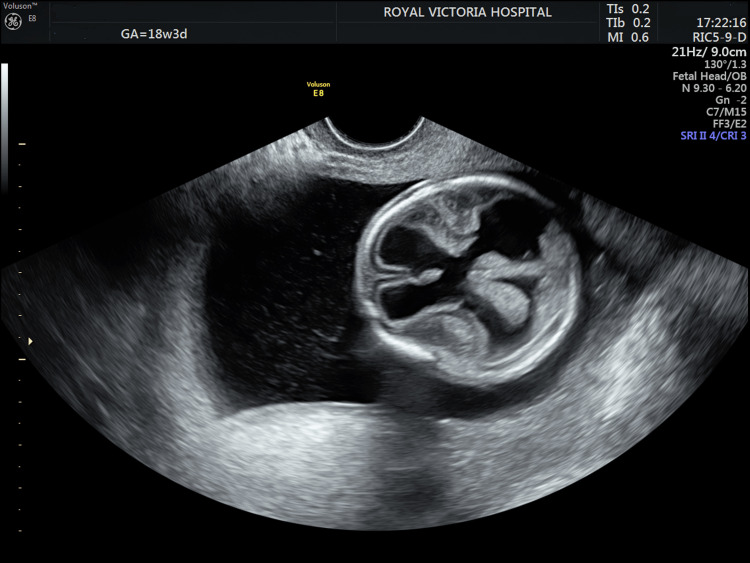
Demonstrating the neurological findings in the affected twin

A 25-year-old primigravida and recent immigrant was referred at 18 weeks' gestation in a spontaneous monochorionic-diamniotic (MCDA) gestation. Ultrasound in her home country at 12 weeks had confirmed monochorionicity, normal nuchal translucencies, and concordant fetal growth. Our initial evaluation at 18 weeks demonstrated a 20% inter-twin growth discordance with no other features of twin-to-twin transfusion syndrome. One fetus had cerebral abnormalities suggestive of a destructive hemorrhagic process. The midline falx was preserved with asymmetric ventricles and abnormal cortical appearances (Figure 1).

Anomalous umbilical venous drainage directly into the inferior vena cava and perihepatic calcifications were also observed; biometry was at the 5th centile. The co-twin had normal anatomy and biometry (15th centile). The poor prognosis, further investigations (including fetal MRI, invasive genetic testing, congenital infection screening, and alloimmune thrombocytopenia evaluation), and management options (including expectant management with or without palliative care depending on the neonatal condition, selective termination, and adoption) were discussed and reviewed further within a multidisciplinary group. The couple did not wish to return to their home country despite being responsible for all healthcare costs.

Amniocentesis of both twins demonstrated normal quantitative fluorescent-polymerase chain reaction (QF-PCR) and chromosomal microarray analysis while whole exome sequencing identified a mutation unique to the abnormal twin in the chromodomain-helicase-DNA-binding protein 7 (CHD7) gene, where autosomal dominance is associated with CHARGE.

Fetal MRI at 19 weeks showed extensive ischaemic changes with additional hemorrhagic transformation affecting the territories of both middle cerebral arteries in the affected fetus. Multidisciplinary counseling reviewed the poor prognosis as well as the impact of in-utero fetal demise upon the co-twin and the various management options were re-discussed. The parents preferred selective termination. However, being responsible for all healthcare costs (including neonatal intensive care-NICU), they asked that reduction be deferred as late as possible to minimize the length of NICU admission. They clearly understood that even with close surveillance the risk of single twin demise and its sequela could not necessarily be avoided. Weekly umbilical Doppler evaluation remained satisfactory in both fetuses as did cerebral and ductus venosus Dopplers in the normal twin. The intertwin discordance remained stable; the normal and affected twins maintained growth at the 15th and 5th centiles respectively. 

Our preferred approach to MCDA selective reduction is RF or microwave cord occlusion, which we have performed until as late as 31 weeks of gestation. Palliative care approaches were reviewed as were the potential difficulties of cord occlusion at advanced gestations with increasing vessel size; despite this and being aware of the continuing risk and consequences of a single fetal demise, the parents maintained their desire to pursue selective feticide only closer to term.

Despite the growth restriction of the affected twin, there were concerns that conventional selective reduction techniques might pose greater challenges in the later third trimester given the size of the cord and fetal vessels, with risks of failed coagulation or vessel rupture and subsequent potential adverse effects on the co-twin. Therefore, alternative approaches were considered and discussed with the parents. We finally proposed selective reduction using an intravascular-occlusion approach rather than an ablative technique, followed immediately by cesarean delivery (both twins being in a transverse lie). An endovascular balloon would be placed at the origin of the aorta occluding both the aorta and coronary arteries with asystole resulting from both cardiac ischemia and vascular occlusion and with the aorta and umbilical arterial circulations being occluded. Institutional approval was obtained to undertake the procedure and the parents consented to what was clearly identified as an experimental technique. 

**Figure 2:**
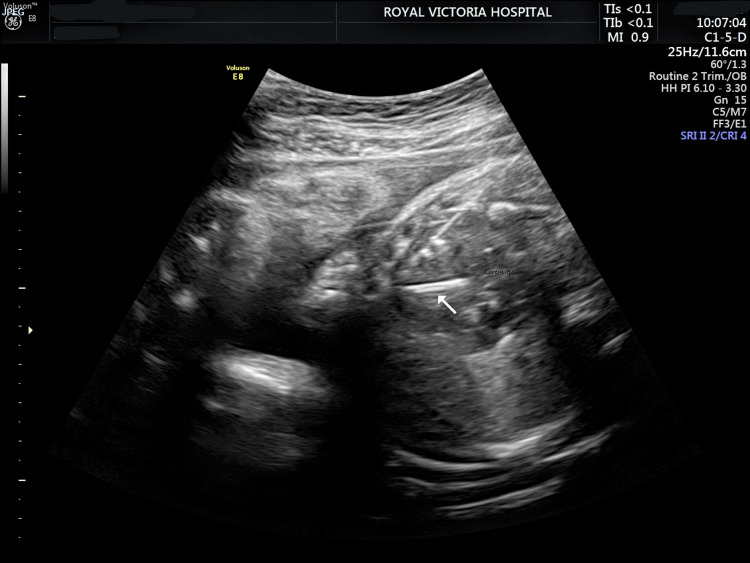
Demonstrating the endovascular balloon (arrow) inflated in the fetal left ventricular outflow

Delivery was planned, with the NICU and to avoid spontaneous labor, at 35 weeks and 4 days gestation (our center normally delivers MCDA twins at 36 weeks), following the administration of betamethasone. Fetal anesthesia (1 mg of Rocuronium and 10 mcg of Fentanyl) was administered intramuscularly under ultrasound guidance to the abnormal twin, with prior maternal combined spinal-epidural anesthesia. The placenta was posterior. A 15-gauge needle was inserted through the anterior fetal thoracic wall into the left ventricle and directed toward the outflow tract. A 6-mm Sterling over-the-wire balloon dilatation catheter (Boston Scientific, Massachusetts, USA) was directed into and through the aortic outflow, and the balloon was dilated with 0.3 ml of saline (Figure 2).

The fetal heart became bradycardic with a progressive reduction in rate until asystole was achieved. The normal twin’s umbilical artery Doppler was periodically evaluated and demonstrated normal flow throughout the procedure, while no flow, arterial or venous, was observed in the abnormal twin’s cord, suggesting no reverse perfusion that might risk the co-twin’s cardiovascular status.

Sustained asystole was observed after a few minutes with no flow in the demised twin’s cord and the balloon was then deflated and the catheter removed. Absence of flow in the demised fetus and its cord and normal flow and heart rate in the normal twin were again observed suggesting no intra-fetal transfusion and cesarean delivery was then immediately performed (stillborn fetus - 1670 g; healthy twin - 2010 g). The healthy twin was born in good condition with arterial and venous cord pH’s of 7.31 and 7.33 respectively. After neonatal evaluation, the newborn did not require NICU admission and was later discharged home with outpatient pediatric follow-up. Development at 3 months was normal. 

The parents did not consent to autopsy or examination of the reduced twin, though placental pathology confirmed monochorionicity with a 35%/65% division of mass between twins 1 and 2; injection studies were not performed. 

## Discussion

Discordant structural anomalies are found in 3% [4] of MCDA pregnancies and with increasing use of next-generation genetic testing, more cases with genomic discordances are also likely to be found [5], perhaps further increasing the indications for selective fetal termination where appropriate. Although the fetal reduction in MCDA twins is well described, all techniques risk miscarriage or prematurity when performed in the pre-viable or pre-term period, with co-twin death occurring in 10-15% [6].

Ablative selective termination techniques have limitations in the size of vessels that can be effectively occluded and therefore upon the gestational age range where they can safely be performed. Most series describe cases performed in the 18-24-week range with very few cases being reported beyond 30 weeks’ gestation [1,4]. Large vessels (>3 mm) impede the efficacy of RF, acting as heat sinks [7,8] and risking failed ablation or vessel rupture. In this case, the target umbilical vein and artery diameters were already 6.9 mm and 3.1 mm respectively at 33 weeks raising concerns regarding ablative efficacy. Therefore, an approach that may avoid the possible limitations of ablative techniques at late gestations, was considered. It was believed that asystole and vascular occlusion could be achieved using endovascular occlusion of the fetal aorta and coronary circulations rather than utilizing a thermal ablation technique. Partial-birth abortion is not undertaken within our practice though may also have been an option in some areas. The approach chosen will certainly not be applicable in all cases for several reasons including the fetal and placental positions, mode of delivery intentions, and should this become necessary on a more emergent basis, for example, due to preterm labor. In addition, placement of such a balloon catheter is technically more challenging than for example placement of an RF antenna at the fetal cord insertion and therefore the experience of available personnel in performing invasive fetal procedures will also guide whether such an approach can even be contemplated. Over-inflation of the balloon can also risk vessel rupture and careful planning and consideration of all the procedure-related needs and complications must be considered beforehand. However, in very select cases where selective termination is indicated and when the risk of co-twin demise during pregnancy appears to be low or the indication for selective reduction is only identified later in gestation, this may offer an alternative approach to permit late selective termination in monochorionic twins to be performed immediately prior to delivery. 

## Conclusions

In current practice, conventional and proven approaches to selective termination in monochorionic twins must always be considered and offered first. In the unusual circumstances when clinical presentations justifying selective termination present late in gestation or when additional factors may influence parental decision making, particularly regarding the timing of selective termination, the technique described here may offer an alternative approach in suitable cases.

Appropriate fetal and placental positioning is important to enable the procedure to be even considered. Parents should also be clearly aware that this is a technique that has not been evaluated in detail. The suitability of this approach with a subsequent vaginal delivery being intended would not only be influenced by obstetrical factors but the necessity for more prolonged confirmation of absent flow in the reduced twin’s cord than was necessary in this case, where caesarean was undertaken immediately upon removal of the balloon catheter. At present this should only be considered as an exceptional approach to this clinical problem and one whose case suitability and more importantly overall safety, particularly with respect to the surviving co-twin, requires further evaluation. Further studies and assessment of this approach in suitable cases will need to be conducted before the safety and efficacy of such an intravascular occlusion technique can be established. Given the relative rarity of suitable cases, such an approach may require multi-centre collaboration.
